# Developing a decision aid to guide public sector health policy decisions: A study protocol

**DOI:** 10.1186/1748-5908-6-46

**Published:** 2011-05-10

**Authors:** Peggy Tso, Anthony J Culyer, Melissa Brouwers, Mark J Dobrow

**Affiliations:** 1 Department of Health Policy, Management and Evaluation, University of Toronto, Toronto, ON, Canada; 2 Cancer Services and Policy Research Unit, Cancer Care Ontario, Toronto, ON, Canada; 3 Department of Clinical Epidemiology and Biostatistics, McMaster University, Hamilton, ON, Canada; 4 Program in Evidence-Based Care, Cancer Care Ontario, Toronto, ON, Canada

## Abstract

**Background:**

Decision aids have been developed in a number of health disciplines to support evidence-informed decision making, including patient decision aids and clinical practice guidelines. However, policy contexts differ from clinical contexts in terms of complexity and uncertainty, requiring different approaches for identifying, interpreting, and applying many different types of evidence to support decisions. With few studies in the literature offering decision guidance specifically to health policymakers, the present study aims to facilitate the structured and systematic incorporation of research evidence and, where there is currently very little guidance, values and other non-research-based evidence, into the policy making process. The resulting decision aid is intended to help public sector health policy decision makers who are tasked with making evidence-informed decisions on behalf of populations. The intent is not to develop a decision aid that will yield uniform recommendations across jurisdictions, but rather to facilitate more transparent policy decisions that reflect a balanced consideration of all relevant factors.

**Methods/design:**

The study comprises three phases: a modified meta-narrative review, the use of focus groups, and the application of a Delphi method. The modified meta-narrative review will inform the initial development of the decision aid by identifying as many policy decision factors as possible and other features of methodological guidance deemed to be desirable in the literatures of all relevant disciplines. The first of two focus groups will then seek to marry these findings with focus group members' own experience and expertise in public sector population-based health policy making and screening decisions. The second focus group will examine issues surrounding the application of the decision aid and act as a sounding board for initial feedback and refinement of the draft decision aid. Finally, the Delphi method will be used to further inform and refine the decision aid with a larger audience of potential end-users.

**Discussion:**

The product of this research will be a working version of a decision aid to support policy makers in population-based health policy decisions. The decision aid will address the need for more structured and systematic ways of incorporating various evidentiary sources where applicable.

## Background

Advances in healthcare and social policy have led to dramatic improvements in health worldwide. However, health systems remain under severe pressure. Prevalent trends among high-income countries, including decreasing economic growth rates, escalating costs, aging populations, and elevated public expectations, feed concerns about sustainability, cost-containment, quality improvement, and accountability [1]. In response to these pressures, governments and health organizations are increasingly relying on evidence of effectiveness, appropriateness and implementability to justify practices and policies. The World Health Organization (WHO) has added further emphasis, highlighting the need to develop mechanisms to support the use of research evidence in creating clinical practice guidelines, health technology assessments, and health policy [2]. Underlying this trend is the positioning of scientific rigour as a means of enhancing the legitimacy and effectiveness of decision-making processes.

Decision aids/support tools (hereafter referred to as decision aids) have been developed in a number of health disciplines to support evidence-informed decision-making. One example is the extensive development of clinical practice guidelines used to influence clinical decision-making (*e.g.*, http://www.guidelines.gov). A recent systematic review in the Netherlands found that evidence-based clinical guidelines helped to improve processes and structures of care and patient health outcomes [3]. Another example relates to patient decision aids, increasingly used as an effective way to improve patients' understanding of treatment options and to incorporate this information into 'shared' clinician-patient decision-making processes. O'Connor *et al. *demonstrated that patient decision aids for those facing decisions concerning cancer screening and treatment have a positive effect in improving patients' understanding of the determinants of decisions (*i.e.*, better knowledge of options, benefits, or risks; more realistic expectations; value-based) [4].

In contrast to the clinical context, decision aids to support health policy processes and structures are less well developed. Policy contexts have different complexities and uncertainties than clinical contexts that require different approaches for identifying, interpreting, and applying various types of evidence to support decisions [5-9]. A recent series of articles edited by Oxman and Hanney contributed to filling this gap within health policy decision making, developing a series of tools to support various aspects of health policy making related to research evidence, from the identification of research evidence needs and the search for and assessment of such evidence to its translation into policy decisions [10]. The tools also brought to light some policy considerations other than research evidence (*e.g.*, values, windows of opportunity, the use of policy dialogues); however, they do not directly provide an explicit approach for assessing and incorporating this non-research evidence into the decision-making process. While this work is comprehensive in its approach to the integration of research evidence, particularly systematic reviews, into policy decisions, the focus remains on research evidence rather than adequately representing all types of evidence in the policy decision.

The proposed study aims to add to the current state of knowledge by focusing on how to support health policy decision making more generally, not only in relation to using research evidence but also to the structured and systematic incorporation of non-research evidence into the policy-making process. Non-research evidence, or colloquial evidence, can be understood as the expertise, views, and realities of stakeholders, including 'evidence about resources, expert and professional opinion, political judgment, values, habits and traditions, lobbyists and pressure groups, and the particular pragmatics and contingencies of the situation' [11]. This proposed study is part of an overarching project that is examining how evidence from various sources, research-based and otherwise, is incorporated into colorectal cancer (CRC) screening policy decisions in five Canadian provincial health systems. Previously conducted key informant interviews with clinical leaders, screening experts, regional/local administrative leaders, and government officials from these five provinces helped to evaluate and compare the policy-making processes (including evidence utilization therein) used in their decisions to (not) implement population-based CRC screening programs. Given a common research evidence-base to inform the provinces' policy decisions, inter-provincial variation was apparent in both policy decision processes and outcomes. The current study seeks to build upon those interview findings in order develop a decision aid to inform a decision to implement a population-based cancer screening program. The decision aid is meant to assist policy makers in thinking through different elements of these complex decisions by providing a comprehensive series of prompts that elicit both research- and non-research-based evidence pertinent to the policy decision. The intent is not to develop a decision aid that will yield uniform recommendations across jurisdictions; however, the decision aid should facilitate more transparent policy decisions that incorporate broader and more appropriate types of evidence. The aid will be targeted for use by policy makers and those supporting them. The former include those with the power to make or influence policy decisions; the latter include those who facilitate by informing those decisions [12]. Recognizing these different roles, the decision aid is not intended for use by any single individual but is meant for the collaborative and interdependent efforts that comprise the policy-making process. While an appropriate governing authority ideally should take responsibility for using the decision aid, it is expected that various individuals and groups with different skills and expertise will be tasked with assessing and contributing the relevant information as highlighted by the decision aid's key components.

Based on the above considerations, this study will address, both descriptively and normatively, the following research questions:

1. What is (should be) the purpose of a decision aid for population-based health policy decisions?

2. How are (should) decision aids for population-based health policy decisions (be) conceptualized and constructed?

3. How are (should) decision aids for population-based health policy decisions (be) operationalized and implemented?

## Methods

The development of the proposed decision aid will be guided by three methods: modified meta-narrative review, focus groups, and the Delphi method.

### Phase one: modified meta-narrative review

A modified meta-narrative review will be used to inform the initial development of the decision aid. Findings of the review will help to identify current and possible domains to be considered in a policy decision aid and various other construction aspects (*e.g.*, information presentation, format of decision aid, *et al.*). Because research on decision aids spans many fields and disciplines and uses diverse terms and definitions, standard systematic reviews are not an ideal approach for reviewing the literature [13]. In contrast, the meta-narrative review method, developed by Greenhalgh *et al. *[14], is better for sorting through a vast, heterogeneous literature encompassing multiple research fields carried out by different scientific communities. Its use of narrative and acknowledgement of different contributing research traditions enables a comprehensive comparison of the literature(s) despite differences in methodology, jargon, criteria for success and quality assessment, and approaches to research questions.

The development of the meta-narrative review method stemmed from a large literature review of the diffusion of innovations [15]. As part of this approach, a large multidisciplinary research team, whose backgrounds spanned the relevant research traditions of interest, was assembled. This was done by seeking collaborations between different institutions and departments in order to provide the appropriate skill mix. In comparison, our proposed meta-narrative review will be led by a single investigator in consultation with five to ten advisors assembled to provide expertise in a range of different fields for guiding the review. The number of advisors will depend on the number of relevant research traditions identified. As noted by Greenhalgh *et al. *[14], the list of key research traditions relevant to the research questions will likely evolve as data emerge through the review process.

An initial exploratory search will be conducted to identify potential research traditions relevant to decision aids and respective experts in related fields (*e.g.*, evidence-based medicine, patient decision aids, shared decision making, knowledge translation/exchange, policy frameworks/tools, *et al.*). This search will be carried out through review of traditional healthcare and non-healthcare indexes (*e.g.*, Medline, Embase, Scholar's Portal, *et al.*), Google searches and consultations with experts in the field. Potential advisors will be formally contacted and invited to participate.

Following the exploratory search, expert advisors will be interviewed individually at two time points. The initial interview will be conducted prior to beginning the formal literature search. The purpose of this interview will be to have expert advisors provide guidance on relevant tradition-specific areas of research (*e.g.*, specific search terms, relevant databases, predominant theoretical bases, *et al.*), and identify seminal articles and prominent concepts or themes to support the search and mapping phases of the review. The investigator will then identify and map articles within each research tradition by searching electronic databases, reviewing reference lists of identified papers, contacting key authors in each tradition, and searching the grey literature. The search will focus on work that explores the development of a decision aid rather than only the use of an aid. Comparable studies will be grouped together along with key findings. The mapping phase will result in a narrative account tracing the historical development of concepts, theory, and methods within each research tradition, referred to as meta-narratives.

In synthesizing the research findings across traditions, key themes or dimensions pertinent to our research question will be identified, along with the contribution(s) of each meta-narrative to it. Divergence between meta-narratives with respect to these themes will be examined for possible theoretical causes arising from the meta-narratives in question. It is at this point that expert advisors will be interviewed a final time, presenting them with working narrative accounts to ensure accurate and thorough interpretation of the literature within each tradition. In concluding the meta-narrative review, overall findings will be summarized and a series of recommendations will be made for its practical application to the development of a decision aid to support evidence-informed public sector population-based health policy decisions. As highlighted by Greenhalgh *et al. *[14], recommendations should be grounded through the context provided by multidisciplinary dialogue and consultation with potential end-users of the review. In this case, the context will be the current policy environment wherein public sector health policy decisions are made on behalf of the population. Thus, the meta-narrative review overlaps and feeds into the next phase of the proposed study, focus groups. Initial findings from the meta-narrative review will be used to create a guide for the first focus group discussion enabling members to reflect and comment on the meta-narrative review findings, given their experiences and expertise regarding high-level health policy making.

### Phase two: focus groups

Two focus groups will be conducted with approximately 10 to 12 members of Canada's National Colorectal Cancer Screening Network (NCCSN). The network acts as a national forum for review, discussion, and action on matters of mutual interest or concern related to CRC screening [16]. Network membership comprises key decision makers (including clinicians and political leaders at provincial and territorial levels) and cancer control community partners across Canada. A presentation of this study has been delivered to members of the NCCSN during their May 2010 meeting, where individual members expressed interest in participating. Members will receive a formal email invitation to participate in the focus group. The invitation will provide further study details, outlining the purpose, methods, and expected findings/deliverables of the research study, expectations for their involvement in the study, potential risks associated with study participation, and the measures that will be taken to ensure the confidentiality of responses.

The objective of the first focus group will be to elicit the expertise and experience of focus group members in public sector population-based health policy making and screening decisions. This will provide context for grounding the recommendations made from the modified meta-narrative review. Discussions will revolve around construction aspects (*e.g.*, information domains, information representation, format of decision aid, *et al.*). Moreover, they will provide guidance as to how these recommendations -- in conjunction with overall findings from the meta-narrative review and key informant interviews from earlier work -- can be applied in the development of the decision aid within the current policy environment. As a working draft of the decision aid is developed based on findings from the previously conducted key informant interviews, the modified meta-narrative review, and the first focus group session, it will be sent to participants in advance of conducting the second focus group. The objective of the second focus group will then be to examine issues of application (*e.g.*, feasibility, usefulness, *et al.*) and inform further refinements to the draft decision aid which will be the focus of the Delphi method.

### Phase three: delphi method

The Delphi method facilitates consensus among a panel of experts through a series of structured questionnaires, known as rounds [17]. We chose this technique as it offers a systematic and interactive approach to eliciting expert and stakeholder opinions (particularly targeting end-users of the decision aid). Further, it provides the advantage of consulting with a larger, geographically diverse and interdisciplinary group than other methods, like the nominal group technique would allow [18]. The objective of this phase of our study is to further inform and refine the decision aid, following changes made according to the focus group feedback.

Because the literature has not established consensus on the appropriate sample size for expert panels [19-21], the main goal was to assemble a purposive sample, representative of major stakeholders within the CRC screening decision-making process. All key informants interviewed as part of the completed stages of the broader study examining evidence utilization in support of CRC screening policy in the five provinces (n = 56) and members of the NCCSN (n = 35) will be invited to participate on the Delphi panel (n = 78 after excluding duplicates). We anticipate that approximately 50 invitees will participate in the panel, based on the interest received at the NCCSN meeting held in May 2010 and the enthusiasm of key informants during previous interviews. Prospective panellists will receive a formal invitation to participate in the Delphi panel. The invitation will outline the purpose, methods, and expected findings/deliverables of the research study, expectations for their involvement in the study, potential risks associated with study participation, and measures that will be taken to ensure the confidentiality of responses. A survey will be created to elicit panellists' expert opinions and experience as to the feasibility, usefulness, and comprehensiveness of the various elements contained within the draft decision aid. In addition, a qualitative component will be included as part of the survey to allow participants the opportunity to discuss and compare the proposed decision aid with current practices and its fit within current policy processes. The survey will be distributed to members of the Delphi panel through a web-based survey tool. After each round, the Delphi panel will be presented with an anonymous summary of the previous round's results, along with noteworthy comments and rationale for judgements from which they fill out the next round of survey. The process will carry on until either consensus among panellists is reached or a point of saturation is achieved where no novel data are collected [22].

## Discussion

In answering our research questions looking at the purpose, development, and operationalization of a decision aid to support population-based health policy decisions, a working version of a decision aid will be produced and will have received preliminary evaluation through the focus groups and Delphi. While the context of our study lies within cancer screening policy decisions, it is our hope that the decision aid will be generalizable to other health policy decisions, which we will target in subsequent research. The decision aid aims to facilitate decision makers in making transparent decisions and addresses the need for more structured and systematic ways of integrating various evidentiary sources where applicable. We believe the study design is appropriate to achieve these aims. The modified meta-narrative review will provide invaluable insights in the creation of the decision aid, particularly because population-based health policy decisions are often made in the context of significant complexity and uncertainty, drawing from a broad array of evidentiary sources and impacting various different policy sectors. Conducting the focus groups and Delphi technique are important steps in developing and refining the decision aid to ensure its appropriateness and implementability in the current policy environment.

## Competing interests

The authors declare that they have no competing interests.

## Authors' contributions

All authors contributed to the conceptualization and design of the proposal. PT wrote the initial draft of the manuscript. All authors critically reviewed and provided substantive comments to it and subsequent drafts, and approved the final manuscript.
